# The role of loneliness in the association between chronic physical illness and depressive symptoms among older adults: A prospective cohort study

**DOI:** 10.1016/j.jad.2023.04.072

**Published:** 2023-08-01

**Authors:** A. Kandola, F. Solmi, O. Ajnakina, E. Ingram, E. Iob, S. Lee, A. Steptoe, T. Wright, G. Lewis

**Affiliations:** a MRC Unit of Lifelong Health and Ageing, University College London, London, UK; b Institute of Mental Health, University College London, London, UK; c Division of Psychiatry, University College London, London, UK; d Department of Biostatistics & Health Informatics, Institute of Psychiatry, Psychology and Neuroscience, King's College London, University of London, London, UK; e Department of Behavioural Science and Health, Institute of Epidemiology and Health Care, University College London, London, UK; f Department of Applied Health Research, Institute of Epidemiology and Health Care, University College London, London, UK

**Keywords:** Loneliness, Depression, Chronic disease, Cohort, Mediation

## Abstract

**Background:**

Chronic physical illness increases the risk of subsequent depressive symptoms, but we know little about the mechanisms underlying this association that interventions can target. We investigated whether loneliness might explain associations between chronic illness and subsequent depressive symptoms.

**Methods:**

We used English Longitudinal Study of Ageing data, a prospective cohort of adults over 50. Our exposure was chronic illnesses (wave two) including arthritis, cancer, diabetes, cardiovascular disease, stroke, and chronic obstructive pulmonary disease. Loneliness scores were a mediator on the short University of California, Los Angeles Loneliness Scale at wave three. Depressive symptom scores (outcome) were measured using the Centre for Epidemiologic Studies Depression Scale (wave four). We examined associations of chronic physical illness with loneliness and depressive symptoms in univariable and multivariable regression models.

**Results:**

Fully-adjusted models included 2436 participants with the depression outcome and 2052 participants with the loneliness outcome. Chronic physical illness was associated with 21 % (incident rate ratio = 1.21, 95%CI = 1.03–1.42) higher depression scores at follow-up. We found no evidence of an association between chronic physical illness and loneliness and therefore did not proceed to analyses of mediation.

**Limitations:**

More prevalent chronic illnesses could have driven our results, such as cardiovascular disease.

**Conclusions:**

Chronic physical illnesses increase the risk of depressive symptoms in older adults. However, we did not find any that chronic physical illnesses were associated with an increased risk of subsequent loneliness. Therefore, interventions targeting loneliness to reduce depression in older adults with chronic physical illness may be insufficient.

## Highlights

• Chronic physical illness increases the risk of depression, but the underlying mechanisms are unclear
• We found that loneliness does not mediate the illness-depression relationship in older adults
• Interventions to reduce depression in older adults with chronic physical illness should target other factors

## 1 Introduction

Depression is a leading cause of global disability and affects around 322 million people worldwide (World Health Organisation, 2017). Depression is common among older adults and there is evidence that rates are rising in this age group (Yu et al., 2020; Jenkins et al., 2016). Despite its high prevalence and burden in older adults, there are few population-based interventions to prevent depressive symptoms in this age group (van Zoonen et al., 2014; Werner-Seidler et al., 2017; Stockings et al., 2016). (National Academies of Sciences Engineering and Medicine, 2020) Several chronic physical illnesses are associated with an increased risk of future depressive symptoms (Huang et al., 2010), including cardio-metabolic diseases, cancer, chronic obstructive pulmonary disease (COPD), and arthritis (Matcham et al., 2013; Hare et al., 2014; Atlantis et al., 2013; Tabák et al., 2014; Pilevarzadeh et al., 2019). However, the mechanisms underlying this association are unclear. The risk of developing these chronic physical illnesses increases with age (Niccoli and Partridge, 2012; Harris, 2019). Understanding the mechanisms linking chronic physical illness with depressive symptoms in older adults could lead to potential targets for preventative interventions. There are various biological mechanisms that could underlie associations between chronic physical illness and depressive symptoms (Tabák et al., 2014). For example, physiological changes such as elevated inflammation and altered cortisol levels could mediate associations between cardiovascular disease and depression, (Penninx, 2017; Haapakoski et al., 2015; Belvederi Murri et al., 2014). However, fewer studies have examined psychosocial factors that could mediate associations between chronic physical illness and depressive symptoms, such as loneliness.

Loneliness refers to the emotional experience of perceiving fewer or insufficient meaningful social relationships than desired (Perlman and Peplau, 1981). Loneliness is distinct from social isolation, which is an objective lack of social connections (McHugh et al., 2017). In the UK, around 18 % to 27 % of older adults report feelings of loneliness (Victor and Yang, 2012; Lee et al., 2021). The *National Academies of Sciences, Engineering, and Medicine* recently highlighted loneliness and social isolation as major public health risks in older adults (National Academies of Sciences Engineering and Medicine, 2020). Evidence suggests that heightened feelings of loneliness are associated with an increased future risk of depressive symptoms (Lee et al., 2020; Nuyen et al., 2020; Erzen and Çikrikci, 2018). There is also evidence that loneliness increases the risk of subsequent chronic physical illnesses (Petitte et al., 2015; Hawkley et al., 2010; Holt-Lunstad et al., 2015; Valtorta et al., 2016). However, there is a lack of research examining whether chronic physical illness is associated with subsequent loneliness (National Academies of Sciences Engineering and Medicine, 2020). For example, a cross-sectional study reported high levels of loneliness in older adults with chronic illnesses but did not examine whether this was due to illness or other factors (Polenick et al., 2021). A chronic physical illness could limit mobility and functioning, and reduce participation in social activities or opportunities for meaningful contact, causing social isolation and loneliness (Del Pozo Cruz et al., 2021). The experience of ongoing treatment or lifestyle changes could also contribute to loneliness, even in the absence of social isolation. Therefore, loneliness is a potential mechanism through which chronic physical health problems increase the risk of depressive symptoms. To our knowledge, no study has examined the extent to which loneliness might explain associations between chronic physical illness and depressive symptoms in later life.

We conducted a prospective cohort study to investigate the association between chronic physical illness and depressive symptoms, and the potential mediating role of loneliness among older adults. We hypothesised that chronic physical illness would be associated with a greater risk of subsequent loneliness. We also expected that loneliness would account for part of the association between chronic physical illness and later depressive symptoms.

## 2 Methods

### 2.1 Study design and participants

We used data from the English Longitudinal Study of Ageing (ELSA). ELSA is an ongoing nationally representative cohort of adults over the age of 50 that began in 2002 (wave one) (Steptoe et al., 2013). ELSA collects socioeconomic, psychological, health, biological, and genetic data through interviews, questionnaires, and nurse visits. The core ELSA sample was recruited from households that participated in the 1998, 1999, and 2001 Health Survey for England, with a household and individual response rate of 70 % and 67 %, respectively. There have been nine waves of data collection over 15 years, with intervals of around two years between waves. Refreshment samples were recruited at waves three, four, and six to ensure that the full age range remained represented. Compared with the national census, ELSA is representative of the non-institutionalised general population aged ≥50 residing in the UK (Steptoe et al., 2013). Wave one included 12,099 participants with a mean age of 65 (SD 10.75). The London Multicentre Research Ethics Committee provided ethical approval for ELSA, and all participants provided informed consent. We started our study at wave two (2004–2005) as this was when ELSA first recorded loneliness in 9432 participants (82.8 % of wave one sample).

### 2.2 Outcome: depressive symptoms

Depressive symptoms were measured at all waves using the short Centre for Epidemiologic Studies Depression Scale (CES-D) (Radloff, 1977). The short CES-D is reliable and valid in population-based samples of older adults (Karim et al., 2015; O'Halloran et al., 2014). It contains eight items on depressive symptoms over the past week with a dichotomised yes or no response. We removed one item on loneliness (‘I felt lonely’) to avoid inflating associations with the loneliness scale, consistent with prior studies (Lee et al., 2020). Total scores on the seven-item CES-D scores ranged from zero to seven. We used total scores at wave four as a continuous outcome to reflect the reality of depressive symptoms as a continuum and maximise statistical power (Royston et al., 2006). We did not use depression scores at wave three as this was the same time point as the mediator (loneliness) and temporality between mediator and outcome strengthened causal inferences.

### 2.3 Exposure: chronic physical illness

Our main exposure was self-reported chronic physical illness at wave two. We chose chronic physical illnesses that were associated with depressive symptoms in existing longitudinal studies, and likely to impact social functioning (Huang et al., 2010; Matcham et al., 2013; Hare et al., 2014; Atlantis et al., 2013; Tabák et al., 2014; Pilevarzadeh et al., 2019), potentially leading to loneliness (National Academies of Sciences Engineering and Medicine, 2020). These included arthritis, cancer, diabetes, cardiovascular disease, stroke, and COPD. Cardiovascular diseases included arrhythmia, myocardial infarction, congestive heart failure, angina, and heart murmur. We created a binary variable indicating the presence or absence of any chronic physical illness due to relatively low numbers in some of the illness categories.

We excluded participants with any existing chronic physical health condition at wave one. This reduced variation in the timing of disease onset and the possibility that loneliness preceded rather than followed the exposure (i.e., reverse causation) to strengthen causal inferences. However, we included people with chronic physical health conditions at wave one in a sensitivity analysis to check whether their exclusions potentially impacted our main findings. These sensitivity analyses included a categorical exposure variable with four levels (no illness at waves one or two, illness at wave one only, illness at wave two only, and illness and waves one and two). For example, a participant who developed arthritis by wave one and diabetes by wave two would still be included in this sensitivity analysis (illness at waves one and two). Physical illness type and multimorbidity could also influence associations with depressive symptoms and loneliness (Huang et al., 2010; Matcham et al., 2013; Hare et al., 2014; Atlantis et al., 2013; Tabák et al., 2014; Pilevarzadeh et al., 2019). We defined multimorbidity as the presence of more than one chronic physical illness at wave two, consistent with previous studies (Van Den Akker et al., 2001). We created an ordinal multimorbidity variable with three levels (no illness, single illness, multiple illnesses) for a sensitivity analysis.

### 2.4 Mediator: loneliness

Loneliness was measured from wave two onwards using the short version of the University of California, Los Angeles Loneliness Scale (R-UCLA) (Hughes et al., 2004). It included three items: “how often do you feel you lack companionship?”, “how often do you feel left out?” and “how often do you feel isolated from others?”. Responses were scored from 1 (hardly ever or never) to 3 (often), with total scores ranging from 3 (lowest) to 9 (highest). The scale has high validity and is internally consistent (Russell, 1982). We used loneliness scores as a quasi-continuous variable at wave three so there was temporality from exposure and outcome to strengthen causal inferences. We entered the scale as a continuous variable as it maximises statistical power (Royston et al., 2006) and more closely represents the reality of loneliness existing on a spectrum than a binary variable.

### 2.5 Confounders

**Fig. 1:**
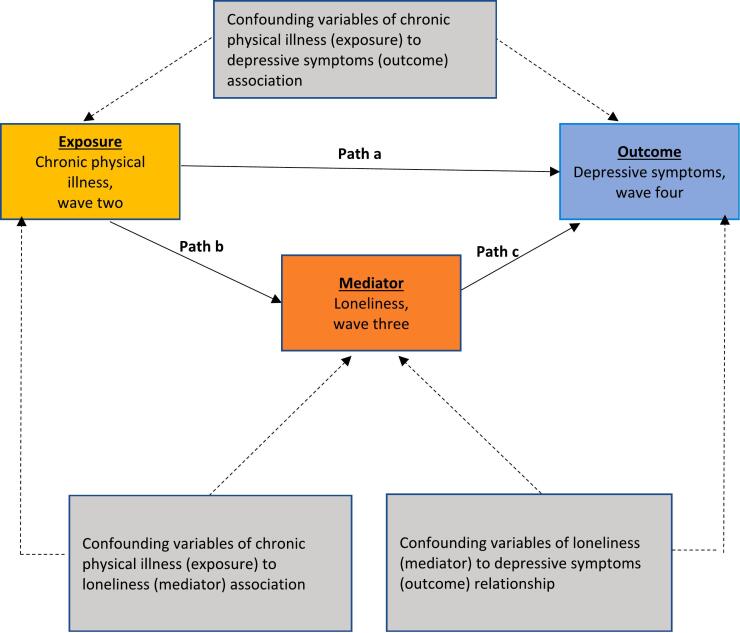
Proposed mediation model.

We selected confounding variables based on previous findings (Lee et al., 2020) and theoretical assumptions, summarized using directed acyclic graphs (Supplementary Materials, Fig. 2). We identified a set of confounders to estimate the direct effect of the exposure on the outcome (path a, Fig. 1) and mediator (path b): age, sex, ethnicity, marital status (unmarried or married or equivalent), education (no, intermediate, or degree and above qualifications), wealth (non-pension wealth in quintiles), employment status (employed or unemployed, including retired), body mass index (BMI) (continuous kilograms/m^2^), physical activity (self-reported frequency of light, moderate, or vigorous intensity physical activity, as categorised in previous ELSA studies (Hamer et al., 2009)), smoking status (never, previous, or current), alcohol use (less than monthly, once or twice a month, once or twice a week, or most days), wave two depressive symptoms (CES-D without loneliness item at wave two), cognitive performance (global cognitive functioning score, including memory, verbal fluency, cognitive speed, attention, and time orientation), polygenic risk scores for depression and loneliness, and wave two loneliness (R-UCLA at wave two). All confounding variables were measured at wave two. See Supplementary Materials Methods 1 for additional details on the measurement and derivation of cognitive performance and polygenic risk scores.

### 2.6 Analysis

We described categorical variables using frequencies with percentages, and continuous variables using means with standard deviations. We describe the models in the analyses below as path *a* or *b* according to Fig. 1. First, we ran traditional regression models to investigate each path as this is recommended as the first step in mediation analyses (Vanderweele, 2015). When assessing path b we used loneliness scores at wave three (hypothesised mediator) as the outcome. If there was evidence of an association, we aimed to assess loneliness scores as a mediator of the association between chronic physical illness (exposure) and depressive symptoms (outcome) using causal mediation analyses based on the potential outcomes framework (Vanderweele, 2015). A previous study has already demonstrated an association of loneliness (mediator) and depressive symptoms (outcome) (path *c*) in ELSA (Lee et al., 2020). The causal assumptions of mediation are that there is evidence of an association between 1) exposure and outcome; 2) exposure and mediator; and 3) mediator and outcome. If we did not find evidence of these associations after all adjustments, we are unable to progress to causal mediation analyses.

### 2.7 Main analysis

We first investigated associations between chronic physical illness and depressive symptoms (path *a*). We used negative binomial regression models with chronic physical illness (wave two) as the exposure and depressive symptoms (wave four) as a continuous outcome. We then investigated path *b* using negative binomial regression models with chronic physical illness as the exposure and loneliness scores (wave three) as a continuous outcome variable. We used negative binomial regression because the discrete depressive symptom and loneliness scores were positively skewed and there was evidence of over-dispersion (see Supplementary Materials, Figs. 4–6 for distributions and dispersion parameters).

We ran univariable, partially and fully adjusted models for path *a* and *b* models. The partially adjusted models contained all confounders except for physical activity, BMI, and baseline versions of the outcome (depressive symptoms for path *a* and loneliness for path *b*). We entered these variables separately as they could mediate rather than confound associations. Adjusting for the wave two loneliness and depression scores reduces the risk of confounding. However, these variables could also be mediators given our assumptions that chronic physical illness may influence loneliness and depressive symptoms. We presented our results with (assuming they are confounders) and without (assuming they are mediators) adjustments for these variables. Negative binomial regression models produce coefficients on the log scale. We exponentiated these coefficients as incident rate ratios (IRR), which are interpretable as percentage changes in depressive symptom or loneliness outcome scores.

Mediation models examine how much of the exposure-outcome association (total effect) is attributable to a mediator (indirect effect). We aimed to establish associations in path *a* and *b*, which we assumed were necessary conditions for mediation. If there was evidence of associations for paths *a* and *b*, we aimed to investigate the full mediation model using causal mediation analysis (Vanderweele, 2015). Causal mediation models operate within a potential outcome framework whereby the aim is to estimate the difference between counterfactual outcomes (i.e., the presence or absence of depressive symptoms) as the averaged causal effect (Vanderweele, 2015).

### 2.8 Sensitivity analyses

We assessed associations of multimorbidity at wave two with depressive symptoms (path *a*, Fig. 1) and loneliness (path *b*, Fig. 1) by repeating models with an ordinal exposure variable based on number of physical conditions. We conducted additional sensitivity analyses to test the robustness of our main findings. These included re-running the main analysis using an exposure variable with four levels that included participants who had an existing chronic physical illness at wave one. We re-ran the path a model with the original short-form CES-D scale with eight items to compare with our modified seven-item scale without the loneliness item. We also re-ran path a and b models after imputing data for missing outcome and confounding variables. We used imputation models as a sensitivity analysis, to check whether missing data due to attrition influenced our main findings. We used multiple imputation models with chained equations to generate 25 datasets. We pooled the results from 25 datasets together with corrected standard errors according to Rubin's rules. The multiple imputation model included all confounding, exposure, and outcome variables used in the main and sensitivity analyses.

## 3 Results

### 3.1 Participant characteristics

We outline the full process of sample selection in Supplementary Fig. 1. We excluded participants with existing chronic physical illnesses reported at wave one (n = 4639 out of 9432), to examine physical illnesses that had begun more recently at wave two, leaving 4793 participants. Of these, 2436 (51 %) had complete data on all variables used to investigate path *a* and 2052 (43 %) path *b*. We provide the descriptive statistics of the sample overall, and according to chronic physical illness at wave two (n = 858). We compared participant characteristics of those included and excluded from our complete case samples in Supplementary Table 1. Arthritis (n = 385 cases) and cardiovascular disease (n = 243 cases) were the most common chronic physical illnesses at wave two, and a full breakdown of illness type is available in Supplementary Materials Table 2. Mean depression scores were 1.11 (SD = 1.59) at wave two, 1.01 (SD = 1.52) at wave three, and 0.95 (SD = 1.49) at wave four. Mean loneliness scores were 3.91 (SD = 1.35) at wave two, 4.00 (SD = 1.42) at wave three, and 4.01 (SD = 1.43) at wave four. Depressive symptom and loneliness scores were moderately correlated at waves two (*r* = 0.37), three (*r* = 0.39), and four (*r* = 0.39) (Table 1).

**Table 1:** Wave two sample characteristics by chronic physical illness.

Characteristic	Overall, N = 4793	No chronic physical illness, N = 3935	Chronic physical illness, N = 858
Sex			
Male	2201 (46 %)	1791 (46 %)	410 (48 %)
Female	2592 (54 %)	2144 (54 %)	448 (52 %)
Marital status			
Unmarried	1135 (24 %)	889 (23 %)	246 (29 %)
Married or equivalent	3657 (76 %)	3045 (77 %)	612 (71 %)
Education			
Higher (degree or above)	707 (15 %)	609 (16 %)	98 (12 %)
Intermediate (school or college qualifications)	2448 (52 %)	2037 (53 %)	411 (50 %)
No formal qualifications	1522 (33 %)	1213 (31 %)	309 (38 %)
Employment			
Unemployed or retired	2662 (56 %)	2057 (53 %)	605 (71 %)
Employed	2082 (44 %)	1837 (47 %)	245 (29 %)
Wealth			
1 (least wealthy)	567 (13 %)	408 (12 %)	159 (20 %)
2	746 (18 %)	590 (17 %)	156 (20 %)
3	909 (22 %)	755 (22 %)	154 (20 %)
4	924 (22 %)	782 (23 %)	142 (18 %)
5 (wealthiest)	1057 (25 %)	886 (26 %)	171 (22 %)
Alcohol use			
Most days	1661 (40 %)	1413 (41 %)	248 (34 %)
Once or twice a week	1138 (27 %)	930 (27 %)	208 (29 %)
Once or twice a month	491 (12 %)	398 (11 %)	93 (13 %)
Less than monthly/none in last year	904 (22 %)	727 (21 %)	177 (24 %)
Smoking			
Previous or current smoker	2806 (60 %)	2286 (60 %)	520 (65 %)
Never smoked	1836 (40 %)	1550 (40 %)	286 (35 %)
Age in years	63 (10)	62 (10)	66 (10)
Total cognitive function score	30 (6)	30 (6)	28 (7)
Body mass index (kg/m^2^)	27.5 (4.6)	27.4 (4.6)	27.8 (4.5)
Wave two depressive symptom score (range 0–8)	1.21 (1.72)	1.13 (1.67)	1.61 (1.90)
Wave two depressive symptom score without loneliness item (range 0–7)	1.11 (1.59)	1.04 (1.54)	1.48 (1.73)
Wave two loneliness score (range 3–9)	3.91 (1.35)	3.89 (1.33)	3.99 (1.47)

Categorical variables are presented as n (%) and continuous variables as means (standard deviation).

### 3.2 Main analysis

We present associations between chronic physical illness and depressive symptoms (path *a*) in Table 2 and associations of chronic physical illness with loneliness scores (path *b*) in Table 3. In the univariable model, there was evidence that having a chronic physical illness was associated with 36 % (IRR = 1.36, 95%CI = 1.18, 1.56) higher depression scores at follow-up, compared with no illness. The fully adjusted models indicated that having a chronic physical illness was associated with a 21 % (IRR = 1.21, 95%CI = 1.03, 1.42) increase in depressive symptom scores at follow-up.

**Table 2:** Associations of chronic physical illness with depressive symptoms (path a).

Model	Reference category	N	Incident rate ratio	95 % confidence intervals	P-value
Univariable	No physical illness	2436	1.36	1.18, 1.56	<0.001
Partially adjusted^a^			1.27	1.06, 1.52	0.008
Partially adjusted^b^			1.24	1.02, 1.49	0.027
Fully adjusted^c^			1.21	1.03, 1.42	0.029

All models use chronic physical illness as the exposure.

a Adjusted for age, sex, marital status, employment status, ethnicity, wealth, alcohol use, smoking status, cognitive performance, polygenic risk of depression, polygenic risk of loneliness, and wave two loneliness score.

b Adjusted for age, sex, marital status, employment status, ethnicity, wealth, alcohol use, smoking status, BMI, physical activity, cognitive performance, polygenic risk of depression, polygenic risk of loneliness, and wave two loneliness score.

c Adjusted for age, sex, marital status, employment status, ethnicity, wealth, alcohol use, smoking status, BMI, physical activity, cognitive performance, polygenic risk of depression, polygenic risk of loneliness, wave two depressive symptom score, and wave two loneliness score.

**Table 3:** Associations of chronic physical illness with loneliness scores (path b).

Model	Reference category	N	Incident rate ratio	95 % confidence intervals	P-value
Univariable	No illness	2052	1.02	0.97, 1.07	0.333
Partially adjusted^a^			1.01	0.95, 1.07	0.852
Partially adjusted^b^			1.01	0.95, 1.07	0.864
Fully adjusted^c^			1.00	0.94, 1.06	0.961

All models use chronic physical illness as the exposure.

a Adjusted for age, sex, marital status, employment status, ethnicity, wealth, alcohol use, smoking status, cognitive performance, polygenic risk of depression, polygenic risk of loneliness, and wave two depressive symptom score.

b Adjusted for age, sex, marital status, employment status, ethnicity, wealth, alcohol use, smoking status, BMI, physical activity, cognitive performance, polygenic risk of depression, polygenic risk of loneliness, and wave two depressive symptom score.

c Adjusted for age, sex, marital status, employment status, ethnicity, wealth, alcohol use, smoking status, BMI, physical activity, cognitive performance, polygenic risk of depression, polygenic risk of loneliness, wave two depressive symptom score, and wave two loneliness score.

We found no evidence of an association between chronic physical illness at wave two and loneliness scores at wave three (Table 3) in univariable (IRR = 1.02, 95%CI = 0.97, 1.07) or fully adjusted models (IRR = 1.00, 95%CI = 0.94, 1.07). Consequently, we did not proceed with the mediation analysis.

### 3.3 Sensitivity analysis

There were 96 (2 %) participants with two or more chronic physical illnesses at wave two, indicating multimorbidity. There was evidence of an association between multimorbidity and depressive symptoms in the univariable model (IRR = 2.04, 95%CI = 1.49, 2.80, n = 2436) but not in the adjusted model (IRR = 1.14, 95%CI = 0.78, 1.66, n = 2436) (Supplementary Table 3). There was no evidence of an association between multimorbidity and loneliness in univariable (IRR = 1.06, 95%CI = 0.93, 1.22, n = 2052) or adjusted (IRR = 0.97, 95%CI = 0.81, 1.15, n = 2052) models (Supplementary Table 4).

Using the exposure variable that includes wave one and two data, compared with no illness at either wave there was an association between illness at wave one only (IRR = 1.25, 95%CI = 1.14, 1.35, n = 4711), illness at wave two only (IRR = 1.21, 95%CI = 1.05, 1.39, n = 4711), and illness at waves one and two (IRR = 1.33, 95%CI = 1.13, 1.56, n = 4711) and depressive symptoms, in adjusted multivariable models (Supplementary Table 5). There was no evidence of an association between loneliness and illness at wave one only (IRR = 1.00, 95%CI = 0.97, 1.03, n = 3887), illness at wave two only (IRR = 1.00, 95%CI = 0.95, 1.06, n = 3887), and illness at waves one and two (IRR = 1.01, 95%CI = 0.95, 1.09, n = 3887) compared with no illness at either wave in adjusted models (Supplementary Table 6).

There was evidence of an association between chronic physical illness and depressive symptoms when using the full CES-D scale (including the loneliness item) in crude (IRR = 1.44, 95%CI = 1.28, 1.62, n = 2436) and adjusted models (IRR = 1.18, 95%CI = 1.04, 1.14, n = 2436). The results of the main analysis were also consistent in full sample models with imputed missing confounder and outcome data (Supplementary Table 7).

## 4 Discussion

### 4.1 Main findings

We conducted a large, prospective cohort study of older adults, to examine the association between chronic physical illness and depressive symptoms, and the potential mediating role of loneliness. We found that depressive symptoms were 21 % higher in people with a chronic physical illness compared to those without. However, we found no evidence of an association between chronic physical illness and subsequent loneliness among older adults. This suggests that loneliness is unlikely to contribute to the association between chronic physical health problems and depressive symptoms. There was no evidence that these associations differed according to physical illness type or the presence of multimorbidity.

Our finding that chronic physical illness was associated with an increased risk of depressive symptoms in older adults is consistent with previous findings including for cardio-metabolic diseases, cancer, COPD, and arthritis (Huang et al., 2010; Matcham et al., 2013; Hare et al., 2014; Atlantis et al., 2013; Tabák et al., 2014; Pilevarzadeh et al., 2019). Interpreting whether effect sizes are meaningful in clinical or public health terms is complex and depends upon several factors including the Minimal Clinically Important Difference and the prevalence of exposure and outcome. Given that depressive symptoms are common in the general population, a relative increase of 21 % in the exposed group is likely to be meaningful. Demonstrating the association with a combined chronic physical illness exposure could indicate shared underlying mechanisms that may increase the risk of depression. The risk of most chronic physical illnesses increases with age (Niccoli and Partridge, 2012; Harris, 2019), and our findings suggest that older adults who receive these diagnoses are at an elevated risk of depressive symptoms. Promoting awareness of this in primary and secondary care could improve prevention, early detection, and treatment of depressive symptoms in older adults.

Our findings indicate that chronic physical illness does not increase the risk of depression among older adults by increasing loneliness in this sample. These results suggest that reducing loneliness among older adults with chronic physical illness may not prevent future depression. However, older adults reporting loneliness may still benefit from interventions to reduce loneliness as it can still cause other adverse outcomes (Lee et al., 2020).

### 4.2 Strengths and weaknesses

Strengths of our study include the large population-based sample that allowed us to assess temporal associations for our proposed mediation model. The repeated measures also allowed for baseline adjustments to reduce the possibility of reverse causation. We tested the robustness of our main findings with several sensitivity analyses, including multiple imputation models to reduce attrition bias. The rich selection of measures in ELSA also allowed us to adjust for a broad range of confounders.

There were several weaknesses of our study. Most of the cases in our sample were either arthritis (n = 385) or cardiovascular disease (n = 243). The experiences of these conditions may have driven our results and limited the inferences we could make about other chronic physical illnesses, such as stroke (n = 44) and COPD (n = 69). This also limited our statistical power for investigating illness type as an effect modifier. Whilst self-reported measures of chronic physical illness could introduce measurement error, evidence suggests that the self-reported prevalence of chronic physical illnesses at wave one in ELSA was comparable with the Global Burden of Disease study estimates for the UK, such as for arthritis (30.1 % versus 31 %) and COPD (5.5 % versus 3.9 %) (Public Health England, 2020). Other factors could also influence how a chronic physical illness might affect feelings of loneliness or depression, such as disease severity, progression, or nature of the treatment.

Measurement error could also have affected our outcome as we used a short CES-D scale rather than clinical interviews. Although self-reported measures reduce observer bias, there may be differences with clinical diagnoses of major depressive disorder. However, self-report measures reduce observer bias. There is also high agreement between self-report and medical record diagnoses for mental and physical health conditions in population-based studies (Bergmann et al., 1998; Simpson et al., 2004). The use of CES-D scales allowed us to assess symptom severity on a continuum, which is a more realistic representation of depressive symptoms. We removed the loneliness item to create a modified version of the CES-D in this study. However, previous studies using this approach have found that the CES-D maintains good internal consistency before and after removing the loneliness item and findings do not differ depending on the presence or absence of this item (Lee et al., 2020).

There was attrition and systematic differences between the sample we used for analyses and the full ELSA cohort may have led to bias. Our main findings were consistent in datasets with imputed data, which indicate that selection bias is unlikely to have substantially affected our models. However, we only imputed data from wave two, and there could still have been selection bias due to the 17 % attrition from wave one. There could also be systematic differences from the Heath Survey for England sample who chose to participate, as ELSA only achieved around a 70 % response rate. There are some situations where selection bias can distort associations. For example, if the exposure (i.e., chronic physical illness) and outcome (i.e., depressive symptoms) influence recruitment or retainment in ELSA, it can introduce spurious correlations when assessing associations between these two variables via collider bias (Munafò et al., 2018). We may have also induced collider bias in our main findings from excluding participants with an existing chronic physical illness from wave one and thereby conditioning on a physically healthier subsample. However, our main findings were comparable when including all wave two participants in a sensitivity analysis model comparing people with and without illnesses at waves one and two.

## 5 Conclusions

We found that chronic physical illness increases the risk of depressive symptoms in older adults, but no evidence that this occurs through loneliness in this study. Healthcare workers in primary and secondary care settings should recognise that older adults who receive chronic physical health diagnoses are at an elevated risk of depressive symptoms and may require additional support. However, interventions targeting loneliness may not reduce depression in older adults with chronic conditions and clinicians and public health professionals should consider other approaches. Future studies could investigate other psychosocial pathways through which chronic physical illness increases the risk of depression, such as the experience of long-term treatments or reduced social participation. Research could also investigate whether chronic illnesses influence loneliness at certain severities, such as impairing physical functioning.

## Funding

Loneliness and Social Isolation in Mental Health Research Network, UKRI. GeL is supported by a Sir Henry Dale Fellowship jointly funded by the 10.13039/100010269Wellcome Trust and the 10.13039/501100000288Royal Society (Grant Number 223248/Z/21/Z).

## Ethics approval and consent to participate

Ethical approval for all the ELSA waves was granted from the National Research and Ethics Committee (MREC/01/2/91), and written informed consent was obtained from all participants. Details of the ELSA study design, sample and data collection are available at the ELSA's project website [https://www.elsa-project.ac.uk/].

## Consent for publication

NA.

## CRediT authorship contribution statement

GL conceptualized the study and all authors contributed to the development of the study design. AK led the analysis and write up. All authors contributed to the final manuscript.

## Conflict of interest

The authors declare no competing interests.

## Data Availability

Data is available through the ELSA's project website [https://www.elsa-project.ac.uk/].

## Appendix A Supplementary data

Supplementary material Image 1

## References

[bb0005] Atlantis E., Fahey P., Cochrane B., Smith S. (2013). Bidirectional associations between clinically relevant depression or anxiety and COPD: a systematic review and meta-analysis. Chest.

[bb0010] Belvederi Murri M., Pariante C., Mondelli V., Masotti M., Atti A.R., Mellacqua Z. (2014). HPA axis and aging in depression: systematic review and meta-analysis. Psychoneuroendocrinology.

[bb0015] Bergmann M.M., Byers T., Freedman D.S., Mokdad A. (1998). Validity of self-reported diagnoses leading to hospitalization: a comparison of self-reports with hospital records in a prospective study of American adults. Am. J. Epidemiol..

[bb0020] Del Pozo Cruz B., Perales F., Alfonso-Rosa R.M., Del Pozo-Cruz J. (2021). Bidirectional and Dynamic Relationships Between Social Isolation and Physical Functioning Among Older Adults: A Cross-Lagged Panel Model of US National Survey Data. J. Gerontol A. Biol. Sci. Med. Sci..

[bb0025] Erzen E., Çikrikci Ö. (2018). The effect of loneliness on depression: a meta-analysis. Int. J. Soc. Psychiatry.

[bb0035] Haapakoski R., Mathieu J., Ebmeier K.P., Alenius H., Kivimäki M. (2015). Cumulative meta-analysis of interleukins 6 and 1β, tumour necrosis factor α and C-reactive protein in patients with major depressive disorder. Brain Behav. Immun..

[bb0040] Hamer M., Molloy G.J., de Oliveira C., Demakakos P. (2009). Leisure time physical activity, risk of depressive symptoms, and inflammatory mediators: the English longitudinal study of ageing. Psychoneuroendocrinology.

[bb0045] Hare D.L., Toukhsati S.R., Johansson P., Jaarsma T. (2014). Depression and cardiovascular disease: a clinical review. Eur. Heart J..

[bb0050] Harris Randall, Harris Randall E. (2019). Epidemiology of Chronic Disease: Global Perspectives.

[bb0055] Hawkley L.C., Thisted R.A., Masi C.M., Cacioppo J.T. (2010). Loneliness predicts increased blood pressure: 5-year cross-lagged analyses in middle-aged and older adults. Psychol. Aging.

[bb0060] Holt-Lunstad J., Smith T.B., Baker M., Harris T., Stephenson D. (2015). Loneliness and social isolation as risk factors for mortality: a meta-analytic review. Perspect. Psychol. Sci..

[bb0065] Huang C.Q., Dong B.R., Lu Z.C., Yue J.R., Liu Q.X. (2010). Ageing Research Reviews.

[bb0070] Hughes M.E., Waite L.J., Hawkley L.C., Cacioppo J.T. (2004). A short scale for measuring loneliness in large surveys: results from two population-based studies. Res. Aging..

[bb0075] Jenkins R., Brugha T., McManus S.P.B. (2016).

[bb0080] Karim J., Weisz R., Bibi Z., ur Rehman S. (2015). Validation of the Eight-Item Center for Epidemiologic Studies Depression Scale (CES-D) Among Older Adults. https://link.springer.com/article/10.1007/s12144-014-9281-y.

[bb0085] Lee S.L., Pearce E., Ajnakina O., Johnson S., Lewis G., Mann F., Pitman A., Solmi F., Sommerlad A., Steptoe A., Tymoszuk U., Lewis G. (2021). The association between loneliness and depressive symptoms among adults aged 50 years and older: a 12-year population-based cohort study. Lancet Psychiatry.

[bb0095] Matcham F., Rayner L., Steer S., Hotopf M. (2013). The prevalence of depression in rheumatoid arthritis: a systematic review and meta-analysis. Rheumatology.

[bb0100] McHugh J.E., Kenny R.A., Lawlor B.A., Steptoe A., Kee F. (2017). The discrepancy between social isolation and loneliness as a clinically meaningful metric: findings from the Irish and English longitudinal studies of ageing (TILDA and ELSA). Int. J. Geriatr. Psychiatry.

[bb0110] Munafò M.R., Tilling K., Taylor A.E., Evans D.M., Smith G.D. (2018). Collider scope: when selection bias can substantially influence observed associations. Int. J. Epidemiol..

[bb0115] National Academies of Sciences Engineering and Medicine (2020).

[bb0120] Niccoli T., Partridge L. (2012). Current Biology.

[bb0125] Nuyen J., Tuithof M., De Graaf R., Dorsselaer Saskia Van, Kleinjan M., Have Margreet Ten (2020). The bidirectional relationship between loneliness and common mental disorders in adults: findings from a longitudinal population-based cohort study. Soc Psychiatry Psychiatr Epidemiol.

[bb0130] O'Halloran A.M., Kenny R.A., King-Kallimanis B.L. (2014). The latent factors of depression from the short forms of the CES-D are consistent, reliable and valid in community-living older adults. Eur. Geriatr. Med..

[bb0135] Penninx B.W.J.H. (2017). Depression and cardiovascular disease: epidemiological evidence on their linking mechanisms [Internet]. Neurosci. Biobehav. Rev. Elsevier Ltd.

[bb0140] Perlman D., Peplau L.A. (1981). Personal Relationships in Disorder.

[bb0145] Petitte T., Mallow J., Barnes E., Petrone A., Barr T., Theeke L. (2015). A systematic review of loneliness and common chronic physical conditions in adults. Open Psychol. J..

[bb0150] Pilevarzadeh M., Amirshahi M., Afsargharehbagh R., Rafiemanesh H., Hashemi S.M., Balouchi A. (2019). Breast Cancer Research and Treatment.

[bb0155] Polenick C.A., Perbix E.A., Salwi S.M., Maust D.T., Birditt K.S., Brooks J.M. (2021). Loneliness During the COVID-19 Pandemic Among Older Adults With Chronic Conditions.

[bb0160] Public Health England (2020).

[bb0165] Radloff L.S. (1977). The CES-D scale. Appl. Psychol. Meas..

[bb0170] Royston P., Altman D.G., Sauerbrei W. (2006). Dichotomizing continuous predictors in multiple regression: a bad idea. Stat. Med..

[bb0175] Russell D.W., Peplau L.A., Perlman D. (1982). Loneliness: A Sourcebook of Current Theory, Research and Therapy [Internet].

[bb0185] Simpson C.F., Boyd C.M., Carlson M.C., Griswold M.E., Guralnik J.M., Fried L.P. (2004). Agreement between self-report of disease diagnoses and medical record validation in disabled older women: factors that modify agreement. J. Am. Geriatr. Soc..

[bb0190] Steptoe A., Breeze E., Banks J., Nazroo J. (2013). Cohort profile: the English longitudinal study of ageing. Int. J. Epidemiol..

[bb0195] Stockings E.A., Degenhardt L., Dobbins T., Lee Y.Y., Erskine H.E., Whiteford H.A. (2016). Psychological Medicine.

[bb0200] Tabák A.G., Akbaraly T.N., Batty G.D., Kivimäki M. (2014).

[bb0205] Valtorta N.K., Kanaan M., Gilbody S., Ronzi S., Hanratty B. (2016). Loneliness and Social Isolation as Risk Factors for Coronary Heart Disease and Stroke: Systematic Review and Meta-analysis of Longitudinal Observational Studies.

[bb0210] Van Den Akker M., Buntinx F., Roos S., Knottnerus J.A. (2001 Jul 1). Problems in determining occurrence rates of multimorbidity. J. Clin. Epidemiol..

[bb0215] van Zoonen K., Buntrock C., Ebert D.D., Smit F., Reynolds C.F., Beekman A.T. (2014). Preventing the onset of major depressive disorder: a meta-analytic review of psychological interventions. Int. J. Epidemiol..

[bb0220] Vanderweele T.J. (2015).

[bb0225] Victor C.R., Yang K. (2012 Jan 1). The prevalence of loneliness among adults: a case study of the United Kingdom. J Psychol Interdiscip Appl [Internet]..

[bb0230] Werner-Seidler A., Perry Y., Calear A.L., Newby J.M., Christensen H. (2017). Clinical Psychology Review.

[bb0235] World Health Organisation (2017).

[bb0240] Yu B., Zhang X., Wang C., Sun M., Jin L., Liu X. (2020). Trends in depression among adults in the United States, NHANES 2005–2016. J. Affect. Disord..

