# Supplementary material for: The role of loneliness in the association between chronic physical illness and depressive symptoms among older adults: A prospective cohort study
Source: J Affect Disord. 2023 Aug 1;334:220–6. doi: 10.1016/j.jad.2023.04.072 (PMC10618404; doi:10.1016/j.jad.2023.04.072)
Supplement: Supplementary file 1 — Supplementary material [file mmc1.docx]

**Supplementary Materials**

**Figure 1. ELSA participant flowchart**

Full ELSA sample = 12,099

Full wave two sample = 9,432

Final sample for analysis = 4,793

Loss to follow-up = 2,667

Excluded participants with a chronic physical illness at wave one = 4,639

Complete exposure, confounder, and depressive symptom data = 2,436

Complete exposure, confounder, and loneliness data = 2,052

**
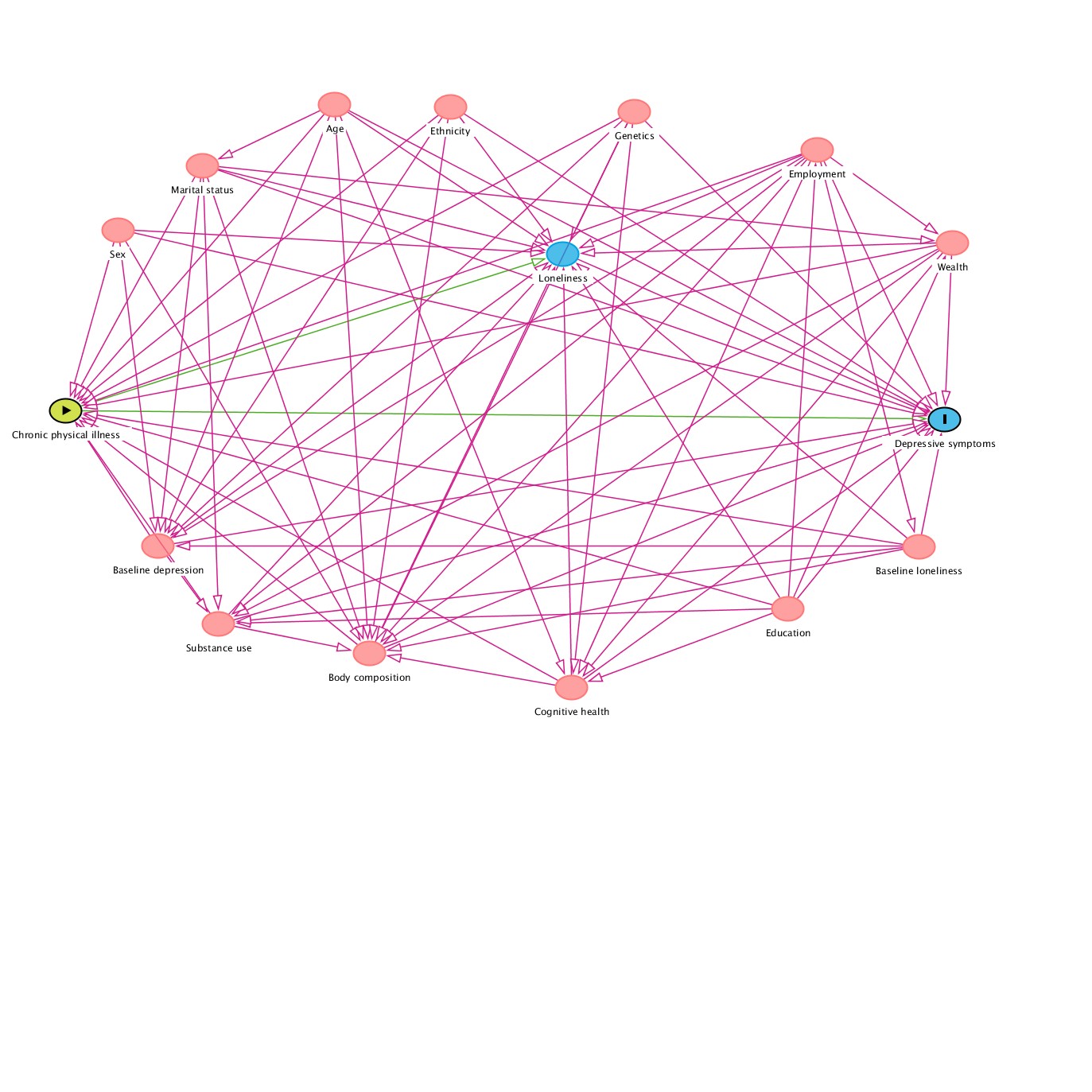
Figure 2. DAG**

*Figure made using dagitty.net*

**Figure 4. Distribution of loneliness scores at wave 3**


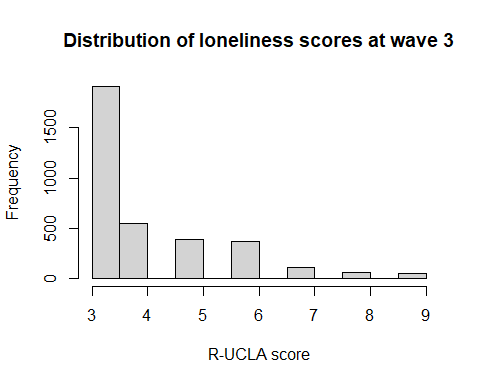


*Dispersion parameter from crude model alpha = 4.88, indicating overdispersion*


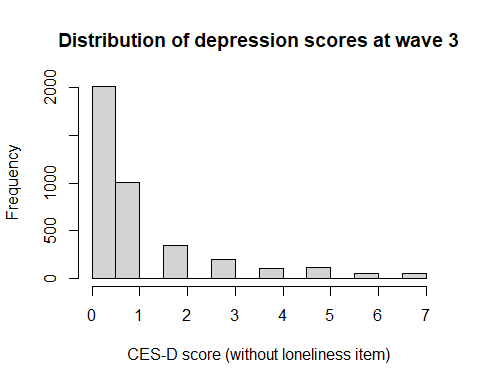
**Figure 5. Distribution of depressive symptom scores at wave 3**

*Alpha = 1.29, indicating overdispersion*


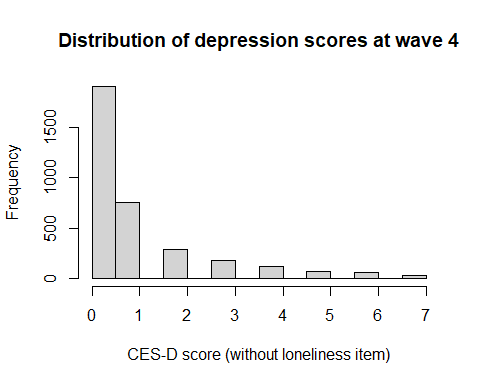
F**igure 6. Distribution of depressive symptom scores at wave 4**

*Alpha = 1.58, indicating overdispersion*

**Methods 1. Cognitive performance and polygenic risk scores**

We used polygenic risk scores to approximate the genetic influence on depression and loneliness as possible confounding variables in our analysis. Full details on the derivation of these polygenic risk scores are available elsewhere (22). The polygenic risk scores use fully genome-wide sequenced data from ELSA. Genome-wide data were available for 7,183 ELSA participants of European ancestry (96.9% of available genomic data 7412) and 1,372,240 single-nucleotide polymorphisms (SNPs) (61.5% of initial an 2,230,767 SNPs) after quality assurance and control procedures (22). The Social Science Genetic Association Consortium (SSGAC) created the polygenic risk scores for depression in our analysis from an initial 6,524,474 SNPs with 1,187,563 SNPs that overlapped with the ELSA genomic data. The Psychiatric Genomics Consortium created the polygenic risk scores for loneliness, using genome-wide data from the Health and Retirement Study of 10,760 people aged 50 or more. Of 5,768,558 SNPs, 1,055,906 overlapped with ELSA genomics data and were included in the polygenic risk score for loneliness. SNPs associated with depression or loneliness were weighted by their effect sizes and summated to a continuous polygenic risk score for each outcome, respectively.

Cognitive performance is assessed in ELSA using a battery of tests for verbal and prospective memory, verbal fluency, cognitive speed, attention, and time orientation. We used a summation of z-scores for each test for a global cognitive function z-score, as in previous studies (22).

**Table 1. Full wave two sample characteristics in by inclusion in analysis**

| Characteristic | Overall, N = 9,432 | Excluded, N = 4,639 | Included, N = 4,793 | *p*-value |
| --- | --- | --- | --- | --- |
| Sex |  |  |  | <0.001 |
| Male | 4,126 (44%) | 1,925 (41%) | 2,201 (46%) |  |
| Female | 5,306 (56%) | 2,714 (59%) | 2,592 (54%) |  |
|  |  |  |  |  |
| Marital status |  |  |  | <0.001 |
| Unmarried | 2,666 (28%) | 1,531 (33%) | 1,135 (24%) |  |
| Married or equivalent | 6,765 (72%) | 3,108 (67%) | 3,657 (76%) |  |
| Education |  |  |  | <0.001 |
| Higher (degree or above) | 1,160 (12%) | 453 (9.8%) | 707 (15%) |  |
| Intermediate (school or college qualifications) | 4,572 (49%) | 2,124 (46%) | 2,448 (52%) |  |
| No formal qualifications | 3,580 (38%) | 2,058 (44%) | 1,522 (33%) |  |
| Employment |  |  |  | <0.001 |
| Unemployed or retired | 6,375 (68%) | 3,713 (81%) | 2,662 (56%) |  |
| Employed | 2,976 (32%) | 894 (19%) | 2,082 (44%) |  |
| Wealth |  |  |  | <0.001 |
| 1 (least wealthy) | 1,583 (18%) | 1,016 (23%) | 567 (13%) |  |
| 2 | 1,724 (20%) | 978 (22%) | 746 (18%) |  |
| 3 | 1,741 (20%) | 832 (19%) | 909 (22%) |  |
| 4 | 1,773 (20%) | 849 (19%) | 924 (22%) |  |
| 5 (wealthiest) | 1,840 (21%) | 783 (18%) | 1,057 (25%) |  |
| Alcohol use |  |  |  | <0.001 |
| Most days | 2,888 (35%) | 1,227 (31%) | 1,661 (40%) |  |
| Once or twice a week | 2,094 (26%) | 956 (24%) | 1,138 (27%) |  |
| Once or twice a month | 983 (12%) | 492 (12%) | 491 (12%) |  |
| Less than monthly/none in last year | 2,179 (27%) | 1,275 (32%) | 904 (22%) |  |
| Smoking |  |  |  | <0.001 |
| Previous or current smoker | 5,858 (63%) | 3,052 (66%) | 2,806 (60%) |  |
| Never smoked | 3,386 (37%) | 1,550 (34%) | 1,836 (40%) |  |
| Age in years | 66 (11) | 69 (11) | 63 (10) | <0.001 |
| Total cognitive function score | 29 (7) | 28 (7) | 30 (6) | <0.001 |
| Body Mass Index (kg/m^2^) | 27.9 (4.9) | 28.4 (5.2) | 27.5 (4.6) | <0.001 |
| Wave two depressive symptom score (range 0-8) | 1.56 (1.96) | 1.94 (2.11) | 1.21 (1.72) | <0.001 |
| Wave two depressive symptom score without loneliness item (range 0-7) | 1.43 (1.78) | 1.76 (1.91) | 1.11 (1.59) | <0.001 |
| Wave two loneliness score (range 3-9) | 4.11 (1.5) | 4.32 (1.36) | 3.91 (1.35) |  |
|  | | | |  |

*Categorical variables are presented as n (%) and continuous variables as means (standard deviation). P-values are from t-tests for continuous variables and chi-squared tests for categorical variables.*

**Table 2. Wave two by illness type**

| Characteristic n (%) | Overall, N = 4,793 |
| --- | --- |
| **Arthritis** |  |
| No case | 4,321 (92%) |
| Case | 385 (8.2%) |
| **Cancer** |  |
| No case | 4,600 (98%) |
| Case | 89 (1.9%) |
| **Diabetes** |  |
| No case | 4,557 (97%) |
| Case | 135 (2.9%) |
| **Cardiovascular disease** |  |
| No case | 4,550 (95%) |
| Case | 243 (5.1%) |
| **Chronic obstructive pulmonary disease** |  |
| No case | 4,621 (99%) |
| Case | 69 (1.5%) |
| **Stroke** |  |
| No case | 4,645 (99%) |
| Case | 44 (0.9%) |

*Cases includes participants with multimorbidity reporting more than one conditions*

**Table 3. Multimorbidity and depressive symptoms**

|  | Model | N | Incident rate ratio | 95% Confidence intervals | P-value |
| --- | --- | --- | --- | --- | --- |
| Univariable | No illness (reference) | 2,436 | - | - | - |
|  | 1 illness |  | 1.35 | 1.20, 1.53 | <0.001 |
|  | >1 illness |  | 2.04 | 1.49, 2.80 | <0.001 |
| Adjusted multivariable*^1^* | No illness (reference) | 2,436 | - | - | - |
|  | 1 illness |  | 1.19 | 1.05, 1.35 | 0.008 |
|  | >1 illness |  | 1.14 | 0.78, 1.66 | 0.502 |

*^1^ Adjusted for age, sex, marital status, employment status, ethnicity, wealth, alcohol use, smoking status, BMI, physical activity, cognitive performance, polygenic risk of depression, polygenic risk of loneliness, wave two depressive symptom score, and wave two loneliness score.*

**Table 4. Multimorbidity and loneliness**

|  | Model | N | Incident rate ratio | 95% Confidence intervals | P-value |
| --- | --- | --- | --- | --- | --- |
| Univariable | No illness (reference) | 2,052 | - | - | - |
|  | 1 illness |  | 1.02 | 0.97, 1.06 | 0.455 |
|  | >1 illness |  | 1.06 | 0.93, 1.22 | 0.341 |
| Adjusted multivariable*^1^* | No illness (reference) | 2,052 |  |  |  |
|  | 1 illness |  | 0.99 | 0.94, 1.05 | 0.728 |
|  | >1 illness |  | 0.97 | 0.81, 1.15 | 0.686 |

*^1^ Adjusted for age, sex, marital status, employment status, ethnicity, wealth, alcohol use, smoking status, BMI, physical activity, cognitive performance, polygenic risk of depression, polygenic risk of loneliness, wave two depressive symptom score, and wave two loneliness score.*

**Table 5. Exposure in full wave two sample with four levels and depressive symptoms**

|  | Model | N | Incident rate ratio | 95% Confidence intervals | P-value |
| --- | --- | --- | --- | --- | --- |
| Univariable | No illness at waves one or two (reference) | 4,711 | - | - | - |
|  | Illness at wave one only |  | 1.70 | 1.58, 1.83 | <0.001 |
|  | Illness at waves two only |  | 1.35 | 1.18, 1.53 | <0.001 |
|  | Illness at waves one and two |  | 2.23 | 1.93, 2.57 | <0.001 |
| Adjusted multivariable*^1^* | No illness at waves one or two (reference) | 4,711 | - | - | - |
|  | Illness at wave one only |  | 1.25 | 1.14, 1.35 | <0.001 |
|  | Illness at waves two only |  | 1.21 | 1.05, 1.39 | 0.009 |
|  | Illness at waves one and two |  | 1.33 | 1.13, 1.56 | <0.001 |

*^1^ Adjusted for age, sex, marital status, employment status, ethnicity, wealth, alcohol use, smoking status, BMI, physical activity, cognitive performance, polygenic risk of depression, polygenic risk of loneliness, wave two depressive symptom score, and wave two loneliness score.*

**Table 6. Exposure in full wave two sample with four levels and loneliness**

|  | Model | N | Incident rate ratio | 95% Confidence intervals | P-value |
| --- | --- | --- | --- | --- | --- |
| Univariable | No illness at waves one or two (reference) | 3,887 | - | - | - |
|  | Illness at wave one only (n = 3,935) |  | 1.08 | 1.06, 1.11 | <0.001 |
|  | Illness at waves two only (n = 848) |  | 1.02 | 0.97, 1.07 | 0.332 |
|  | Illness at waves one and two (n = 654) |  | 1.19 | 1.13, 1.24 | <0.001 |
| Adjusted multivariable*^1^* | No illness at waves one or two (reference) | 3,887 | - | - | - |
|  | Illness at wave one only (n = 3,935) |  | 1.00 | 0.97, 1.03 | 0.927 |
|  | Illness at waves two only (n = 848) |  | 1.00 | 0.95, 1.06 | 0.925 |
|  | Illness at waves one and two (n = 654) |  | 1.01 | 0.95, 1.09 | 0.690 |

*^1^ Adjusted for age, sex, marital status, employment status, ethnicity, wealth, alcohol use, smoking status, BMI, physical activity, cognitive performance, polygenic risk of depression, polygenic risk of loneliness, wave two depressive symptom score, and wave two loneliness score.*

**Table 7. Multiple imputation models**

| Model | Reference category | N | Incident rate ratio | 95% Confidence intervals | P-value |
| --- | --- | --- | --- | --- | --- |
| Path a models (depressive symptoms) | | | | | |
| Univariable | No illness | 4,793 | 1.40 | 1.23, 1.60 | <0.001 |
| Fully adjusted multivariable^1^ |  |  | 1.15 | 1.01, 1.31 | 0.034 |
| Path b models (loneliness) | | | | | |
| Univariable | No illness | 4,793 | 1.03 | 0.99, 1.07 | 0.087 |
| Fully adjusted multivariable ^2^ |  |  | 0.99 | 0.95, 1.03 | 0.663 |

*^1^ Adjusted for age, sex, marital status, employment status, ethnicity, wealth, alcohol use, smoking status, BMI, physical activity, cognitive performance, polygenic risk of depression, polygenic risk of loneliness, wave two depressive symptom score, and wave two loneliness score.*

*^2^ Adjusted for age, sex, marital status, employment status, ethnicity, wealth, alcohol use, smoking status, BMI, physical activity, cognitive performance, polygenic risk of depression, polygenic risk of loneliness, wave two depressive symptom score, and wave two loneliness score.*
